# Advertising One’s Profession and Proprietary Remedies

**Published:** 1896-02

**Authors:** 


### ADVERTISING ONE'S PROFESSION AND PROPRIETARY REMEDIES.

THE long-tolerated custom of English veterinarians placing on the markets proprietary remedies and cure-alls for the relief and cure of all the various groups of disease equine flesh is heir to, and leading to the endless distribution of placards, testimonials, and circulars to increase the sale thereof, has culminated, as it was destined to, in lowering the status of the profession, and bringing down upon its followers' heads deep and severe criticism. The establishment of a rigid code of ethics is now demanded as the only avenue of relief, and to cut out the deep sore from the body professional. Its rigidity seems severe to a fault. Why a veterinarian should not be allowed to insert his card stating his name, profession, location, office hours, telephone number, in any reputable newspaper, we are at a loss to appreciate or understand.

